# Annotations

**Published:** 1905-07-01

**Authors:** 


### ANNOTATIONS.

### Starvation Salaries for Medical Men.

The appointment of two new medical officers to districts of the Guildford Union suggested to some of the Guardians the idea that the time had come to effect economy by the reduction of salaries which they consider extravagant. It appears that the original proposal was to pay one medical officer a salary at the rate of £45 a year, and the other a salary of £20 with the additional appointment of public vaccinator. The Guardian who led the opposition to this scale of remuneration on the ground that it was excessive, urged that in the first case the medical officer would receive from a deposit friendly society proportionately only £13 16s. for the same work, while the suggested conditions of the second appointment he described as an "even more flagrant example of extravagance." The comparison between the salary of a Poor-law medical officer and the fees of a medical man whose services are retained by a friendly society is absurd, because the conditions are altogether different. Nor were the Guildford Guardians deterred by any of the other arguments advanced by the champions of economy from confirming the appointments at the salaries named. So far, they are entitled to credit. But we cannot congratulate them upon their liberality. A great deal is said and written about the miserable stipends of some of the clergy. But even the most indifferently paid curate is not asked to discharge his various ministerial obligations, to hold himself in readiness at all hours of the day and night, and to travel from village to village at his own expense, for less than £1 a week. We assert that remuneration on the scale considered adequate by the Guildford Guardians, whom we only cite as the latest offenders who have come under our notice, ought to be regarded as an insult to the members of a skilled profession. That this view is not adopted is due to the fact that the supply of medical men is greater than the demand. But even that is not a sufficient reason why mere starvation salaries should be offered.

### The Status of Infirmary Patients.

That a man should commit suicide simply and solely because, having been a patient in a poor-law infirmary, he was technically a "pauper," is hardly credible. This an unfortunate councillor of West Ham is said to have done, but it may be presumed that the illness which caused him to be taken to the infirmary, in addition, perhaps, to private troubles, had upset the balance of his mind before he took his own life. Yet, making every allowance for the personal equation, it must be admitted that the loss of status involved in accepting medical treatment in a poor-law infirmary is a humiliation, and in many cases an undeserved humiliation, to those who receive it. The assumption on which it is based is that the infirmary patient belongs to a lower grade socially and even morally than the hospital patient. If this was ever true universally, it is so no longer, and with every improvement in the equipment of infirmaries the difference between them and the voluntary hospitals tends to grow less. That an infirmary patient need not really be a pauper—using the word in its accurate signification of a destitute person—is evidenced by the fact that guardians can and often do reclaim from patients and their friends the cost, or part of the cost, of their treatment in the infirmary. In districts where no voluntary hospital exists it is almost a matter of course that patients who require hospital treatment should be taken to the union infirmary. This state of affairs is a comparatively recent one, or doubtless something would have been done to remove the disabilities of infirmary patients. Some objection might arise on the ground that while they are regarded as indoor paupers each patient earns a certain rebate from the Metropolitan Common Poor Fund, but we do not think that this would prevent many Boards of Guardians from removing from their infirmary patients a stigma which in many cases is undeserved.

### The Metropolitan Water Board.

A letter from Mr. Brudenell Carter, which appeared in the *Times* of June the 23rd, called attention to a very serious default on the part of the Metropolitan Water Board, through which a large area in the south-western suburban district was totally deprived of its customary domestic water supply for a period of about 24 hours. Before the Water Board was constituted we used to hear continual objurgations levelled at the pre-existing companies. Whenever an alleged "famine" occurred, it was the practice of the company concerned to give ample notice of the probable approach of scarcity, to urge the necessity for economical consumption, and to fix temporary standpipes in suitable localities, from which residents near them could obtain water at specified hours. We were led to expect that all this would be altered by the Board, and so it has been, but hardly in the manner anticipated. On the recent occasion no notice of any sort was given to consumers, and no one knew when the supply to the cisterns ceased. The fact that it had ceased was only revealed when the taps no longer made any response to the calls upon them, and this event naturally occurred at somewhat different times in different households. Except for slight variations in the period thus occasioned, the residents in the large districts mentioned were left wholly without water for about 24 hours. Washing, cooking, drinking, bathing, and the flushing of closets were all rendered matters of impossibility for a population of perhaps half a million. If such a failure had occurred under the authority of a company, a summons could have been issued although the highest of the penalties imposed by the old Acts could only be sued for by a public authority. Under the Act constituting the present Board, the penalties of the Water Companies Acts, although possibly left by implication, were not specifically reimposed; so that a magistrate might reasonably doubt his power to inflict them, and his decision, whatever it was, would be liable to be called in question by the High Court. When the possible effects of deprivation of water are considered, especially upon the public health, it may be that the protection now given to the public will have to be increased by fresh legislation, unless the Water Board speedily improves its organisation.

